# Occupational Sun Exposure and Melanoma Development: A Review of the Evidence

**DOI:** 10.1111/ajd.70069

**Published:** 2026-03-02

**Authors:** David C. Whiteman, Gabrielle J. Williams, John F. Thompson

**Affiliations:** ^1^ Department of Population Health QIMR Berghofer Medical Research Institute Brisbane Queensland Australia; ^2^ School of Public Health University of Queensland Brisbane Queensland Australia; ^3^ Melanoma Institute Australia The University of Sydney Sydney New South Wales Australia; ^4^ Faculty of Medicine and Health The University of Sydney Sydney New South Wales Australia; ^5^ Royal Prince Alfred Hospital Sydney New South Wales Australia; ^6^ Faculty of Health and Medical Sciences University of Western Australia Perth Western Australia Australia

**Keywords:** chronic sun exposure, melanoma, occupational exposure, sunlight, ultraviolet light exposure

## Abstract

Cutaneous melanomas arise through a number of causal pathways, all of which involve exposure to sunshine or artificial ultraviolet (UV) radiation to some extent. Their initiation also depends on host factors that determine susceptibility to melanoma, and whether the UV exposure is intermittent or chronic. The question of whether occupational sun exposure causes melanoma has important implications for employees, employers and litigants. This review assessed the evidence linking melanoma development to occupational exposure to sunlight. Electronic literature searches of Medline, Embase and Cochrane CENTRAL to 6 May 2025 were conducted using terms for occupational solar exposure and melanoma. The reviewed observational studies of occupational sun exposure and melanoma development reported both increased and reduced risk. Many of these studies had limited sample sizes and few considered important details such as anatomical site and age, preventing exploration of features that might explain the differing results. The most robust pooled analysis suggested a 45% increase in the risk of melanoma with occupational exposure when studies included the lentigo maligna melanoma (LMM) subtype (relative risk (RR) 1.45, 95% CI 1.08–1.94). When studies excluded LMM the risk of melanoma was reduced (RR 0.69, 95% CI 0.55–0.86). Very few studies have considered occupational sun exposure in the context of the acknowledged causal heterogeneity for melanoma, with the role of chronic sun exposure now understood to differ by anatomical site of tumour, age at diagnosis, and other features. Large‐scale cohort studies reporting risks for the various melanoma subtypes and stratifying for other important risk factors are required. Until the results of such studies are available the contribution of occupational sun exposure to melanoma development in any given patient will continue to require considered, personalised assessment.

## 1 Introduction

For those who are required to work outdoors, the possibility that their risk of developing melanoma and other forms of skin cancer may be increased as a result of their occupational sun exposure needs to be understood. That risk clearly has important implications not only for the individual but also for employers and for those who provide insurance for workers. In Australia and around the world, medico‐legal claims are being made with increasing frequency by outdoor workers who have developed melanomas and keratinocyte cancers. Although there is strongly suggestive evidence, it has to date remained unproven by rigorous scientific assessment that occupational solar exposure increases the risk of cutaneous melanoma, over and above the risk that the individual incurs by exposure to the sun in the course of normal everyday activities and recreational pursuits. Against this background, we sought to critically review all available evidence relating to the impact of occupational exposure to the sun on the risk of developing melanoma.

## 2 The Causal Origins of Cutaneous Melanoma

An association between melanoma development and UV exposure, both from sunshine and from artificial sources, is well‐established. However, our understanding of the way in which various patterns of sun exposure (i.e., chronic, intermittent or total) initiate the development of melanoma has evolved considerably over recent decades. Data reported up to the mid‐1970s gave rise to the hypothesis that the major environmental factor responsible for melanoma development was intense, intermittent exposure to the sun rather than regular, continuous exposure such as that typically received by outdoor workers [1].

Following an intense period of epidemiological inquiry in the 1980s, a number of ‘dual pathway’ models for melanoma initiation were postulated [2, 3, 4]. These variously proposed that melanoma develops through two or more distinct pathways which have different associations with sun exposure, occur at different body sites, and arise at different ages. Further research led to important refinements of these theories, with a new ‘divergent pathway’ model postulating that most cutaneous melanomas are initiated by sun exposure in early life, after which further progression to melanoma depends upon host factors related to the proliferative capacity of the individual's melanocytes [5]. Among people prone to develop benign naevi (which had been shown to be a highly heritable trait), melanoma development was postulated to be driven largely by host factors, perhaps enhanced by intermittent exposure to intense sunlight [6]. Among people ‘resistant’ to developing naevi, melanoma development was postulated to arise following repeated and prolonged exposure to the sun. The two pathways to melanoma development became known as the naevus‐associated pathway and the *de novo* pathway.

These emerging theories aligned with findings from more detailed analyses of population data, which confirmed strikingly consistent patterns of melanoma incidence by age and anatomical site. Hence descriptive studies from the USA [7], Canada [8], Australia [9], New Zealand [10] and the UK [11] showed that among younger people (< 50 years), melanoma density per unit area of skin surface was highest on intermittently sun‐exposed sites (such as the trunk), whereas among older people (50+ years), melanoma density was highest on maximally sun‐exposed sites.

Subsequent molecular studies using genomic sequencing have since confirmed the existence of multiple causal pathways for cutaneous melanoma. These confirmatory genomic findings culminated in the most recent WHO Classification of Skin Tumours (4th edition, 2018) recognising two major pathways for melanoma development, characterised in the new nomenclature as ‘high and low chronic sun damage’ (or ‘high CSD’ and ‘low CSD’). Of special relevance here, it is the class of ‘high CSD’ (or *de novo*) melanomas that are most likely to be attributed to occupational patterns of chronic sun exposure.

## 3 Epidemiological Investigations of Occupation and Melanoma

For this narrative review we identified studies through electronic searches of Medline and Embase (to 5 June 2025), using MeSH headings plus text words ‘melanoma’, ‘occupational exposure’, ‘sunlight’ and ‘ultraviolet rays’ and text words ‘chronic sun exposure’, ‘chronic exposure’ and ‘ultraviolet radiation exposure’. Included studies were those that reported the risks of melanoma in patients with and without occupational sun exposure (see Table 1).

**TABLE 1:** Studies reviewed for assessing occupational sun exposure and melanoma.

First author	Year	Design	No. of studies^a^	Risk of melanoma (95% CI)	Adjusted risks pooled	Heterogeneity measure
Slavinsky [12]	2024	SR	Not stated	No estimate	—	—
Maduka [13]	2023	SR	14	No estimate	—	—
WHO [14]	2021	SR‐MA	**19 (All)**	**RR, All; 1.16 (0.91, 1.49)**	All adjusted	*I* ^2^; 81% (high)
			14	LMM included; 1.45 (1.08–1.94)		*I* ^2^; 81% (high)
			5	LMM excluded; 0.69 (0.55–0.86)		*I* ^2^; 4% (low)
Jiang [15]	2015	SR	10	No estimate	—	—
Chang [16]	2009	MA	15	**OR; H&N, low latitude; 1.7 (1.0, 3.0)**	All adjusted	‘Relative heterogeneity’; 0.05 (low)
				H&N, high latitude; 1.2 (0.8, 1.7)		
				Limbs, low latitude; 1.1 (0.7, 1.6)		
				Limbs, high latitude; 0.8 (0.7, 1.1)		
				Trunk, low latitude; 0.8 (0.5, 1.3)		
				Trunk, high latitude; 1.0 (0.7, 1.3)		
				All sites, low latitude; 1.1 (0.8, 1.5)		
				All sites, high latitude; 0.9 (0.7, 1.1)		
Gandini [17]	2005	SR‐MA	40	RR; 0.95 (0.87–1.04)	Mix of adjusted and crude	*χ* ^2^; 96.06 (high)
Ellwood [18]	1997	SR‐MA	20	OR; 0.86 (0.77–0.96)	Mix of adjusted and crude	*χ* ^2^; 69.7 (high)
Nelemans [19]	1995	SR‐MA	15	OR; 0.73 (0.60–0.89)	Mix of adjusted and crude	*χ* ^2^; 6.12‐population based studies (not high) *χ* ^2^; 30.17‐hospital based studies (high)

Abbreviations: H&N, head and neck; LMM, lentigo maligna melanoma; MA, meta‐analysis; OR, odds ratio; RR, risk ratio; SR, systematic review; SR‐MA, systematic review with meta‐analysis.

^a^ Studies may report intermittent sun exposure as well as occupational or chronic sun exposure but only occupational studies are listed in this table.

### 3.1 Systematic Reviews

We identified six systematic reviews that had assessed the relationship between occupational sun exposure and melanoma risk [13, 14, 15, 17, 18, 19], four of which were published before the 2018 WHO melanoma pathway classification. Two of these reviews, analysing 20 studies [18] and 15 studies [19] respectively, reported a significant reduction in the risk of melanoma for outdoor workers with pooled odds ratios (ORs) of 0.73 [19] and 0.86 [18]. Both these reviews analysed a mix of estimates from crude and adjusted analyses, and both reported substantial heterogeneity. One review aimed to limit heterogeneity by excluding four studies with high risk estimates [18], while the second explored whether melanoma subtype LMM, study blinding, the presence of naevi, and the types of controls influenced levels of heterogeneity [19]. The results of these sub‐analyses showed the greatest consistency for risk of melanoma in outdoor workers when population‐based controls were used and a tendency for studies that included cases of LMM to have more consistent results than when LMM cases were excluded.

A later systematic review that included all the studies from the two previous reviews plus 18 additional studies (resulting in a total of 40 studies) reported a pooled risk estimate for outdoor workers of 0.95 (95% CI 0.87–1.04) but with very high heterogeneity (*χ* ^2^ 96.06, *p* < 0.001), suggesting that the pooled estimate did not well summarise the disparate individual results [17]. Additionally, this review was found to have analysed five risks that were in the opposite direction from those shown in the two earlier systematic reviews and the primary studies, suggesting that the authors had not identified the correct reference group.

Two reviews that included fewer primary studies than prior systematic reviews and did not conduct meta‐analyses provided little information to assist in answering the question of whether melanoma risk is increased by occupational sun exposure [13, 15].

A systematic review conducted by a WHO working group [14] appears to be the most rigorous and informative of the previous reviews that were identified. This review analysed only adjusted ORs and conducted pooled analyses only on studies with very similar comparators (any or high occupational exposure vs. none or low occupational exposure). The review, meta‐analysing data from 19 case–control studies, reported a significantly increased risk of melanoma with occupational sun exposure (summary OR 1.16, 95% CI 0.91–1.49) but with high heterogeneity (*I* ^2^ 81%). This review included a ‘leave‐one‐out’ analysis to measure the effect on heterogeneity and concluded that no single study greatly reduced or increased heterogeneity and that, regardless of the studies analysed, the risk estimates always remained increased for occupational sun exposure. The authors also conducted a sensitivity analysis to assess whether including studies with cases of LMM had a bearing on the estimate; meta‐analysis of LMM‐inclusive studies gave a summary OR of 1.45 (95% CI 1.08–1.94), whereas meta‐analysis of studies that excluded LMM cases gave a summary OR of 0.69 (95% CI 0.55–0.86). The WHO review also included three case–case studies, where cases of melanoma were compared to people with other diseases but who did not have melanoma. All three showed a higher risk of melanoma with occupational sun exposure, but in two of the three, the increase in risk was not statistically significant. One population‐based cohort study was reported in which occupational sun exposure was associated with a 10% increased risk of melanoma, although this was not statistically significant (RR 1.1, 95% CI 0.8–1.6). While the WHO review was robust in its methods, it was limited by the size, quality and reported features within the primary studies.

Finally, and while not a systematic review, a pooled analysis of 15 studies with 5578 melanoma cases and 7024 controls [16] found that occupational sun exposure was associated with an increased risk of melanoma on the head and neck at lower latitudes (pooled OR 1.7, 95% CI 1.0–3.0) but not at higher latitudes (pooled OR 1.2, 95% CI 0.8–1.7) and was not associated with an increased risk of melanoma on the trunk and limbs regardless of latitude.

### 3.2 Limitations of Prior Reviews

There remain fundamental challenges in interpreting the findings of previously‐published systematic reviews that have investigated the possible association between occupational sun exposure and cutaneous melanoma. These challenges may be summarised as (i) understanding heterogeneity, (ii) adequacy of exposure measurements, (iii) control of confounding, (iv) selection bias, and (v) statistical power.

The two systematic reviews that tested for it did identify substantial heterogeneity of effects, and the authors of these reviews made substantial efforts to control for the causes. However, all of the prior reviews relied on primary datasets that reported their findings before the adoption of modern theories of melanoma development (as codified in the 2018 WHO Melanoma Classification). As a result, none of the primary studies, nor the subsequent systematic reviews, compared low‐CSD vs. high‐CSD melanomas (or similar concepts such as ‘naevus‐associated’ vs. *de novo* melanomas). In other words, the reviews and the primary studies on which their data were based have mostly—with a few exceptions—treated all cutaneous melanomas as a homogeneous group of tumours. This approach is likely to have contributed to much of the heterogeneity that has been observed in reported studies.

The second major challenge has been to determine how to accurately measure occupational exposure to sunlight. In small case–control studies it has been possible to collect and analyse detailed information about past sun exposure, albeit with concerns about reproducibility as well as possible biased recall among melanoma cases. Further, very few studies have measured occupational vs. nonoccupational sun exposure and clothing coverage for specific anatomical sites, yet measures of both exposure and coverage are necessary for estimating the true dose of solar radiation to target cells in the skin. In large‐scale studies, detailed exposure measurements have not been possible and authors have generally relied on measurement at a single time point of a generic job description, with all the uncertainty thereby entailed.

The third important issue has been how to control for the effects of confounding factors. Primary among these is the matter of how to disentangle the effects of occupational sun exposure from the effects of nonoccupational exposure. The latter include exposures incurred recreationally (such as when on vacation or when at the beach or playing outdoor sport) and those incurred incidentally (during activities of normal daily living such as shopping, hanging out washing, mowing the lawn, waiting at a bus‐stop). No consensus has been achieved in relation to this substantial question, yet it will have a major bearing on how findings are to be interpreted. Similarly, how should phenotypic factors (such as light skin, inability to tan, proclivity to developing naevi) be handled in these analyses? Are these confounding factors, or are they mediating factors? Again, no consensus exists and primary studies have variously reported effect estimates adjusting for different groups of potential confounders.

The fourth issue that must be considered, especially in lower latitude locations like much of Australia, is the role of selection bias, whereby people with inherently higher risks of melanoma due to their phenotype (e.g., fair skin, tendency to sunburn) are known to self‐select to have indoor occupations [20]. This introduces a major problem for interpretation which cannot be addressed by study design.

The fifth challenge relates to statistical power due to the small numbers of both cases and controls in the groups considered to have high occupational sun exposure (however defined), often representing less than 10% of each study sample. At an overall level, a small sample leads to imprecision in risk estimates (quite apart from the issues of measurement, confounding, bias, and heterogeneity identified above), but also precludes more informative approaches such as stratification and mediation analyses to explore likely associations in a more informed manner.

## 4 Synthesis and Implications

It is well established that most cutaneous melanomas are caused by sun exposure, but the amount and type of exposure required to initiate melanoma development varies according to the susceptibility of the host and the anatomical site of the target cell [21, 22]. This causal complexity is reflected in the bimodal age distributions of melanoma incidence consistently observed in populations of European ancestry around the world, whereby melanomas arising on habitually sun‐protected sites (such as the trunk and, to a lesser extent, the limbs) reach a peak in incidence in the middle decades of life, whereas melanomas arising on habitually sun‐exposed sites (such as the face, ears, neck and male scalp) peak at older ages [7, 8, 9, 11, 23, 24]. These age‐specific and site‐specific incidence patterns also differ by sex [25, 26].

None of the systematic reviews investigated whether the effects of occupational sun exposure (however measured) differed by age, sex or anatomical site. This shortcoming reflects the paucity of primary data on which the systematic reviews were based, since only a handful of studies of any epidemiological design (e.g., ecologic, case–control, cohort, etc.) have accounted for anatomic site when investigating the effects of occupational sun exposure. Arguably the most informative data on this topic stem from an early series of ecological studies using linked occupational and cancer registration data, since they have established temporality, had large sample sizes, and explicitly investigated and reported effects by anatomical subsite.

A correlational study of melanoma notifications in England and Wales reported that outdoor workers had an excess of melanomas of the face, head and neck (+9%) and a significant deficit of melanomas (−22%) at other body sites when compared with the general population [27]. In contrast, office workers were found to have a significant excess of melanomas (+31%) at sites other than the face, head and neck. A Swedish study of similar design reported similar findings [28]. Another study using record linkage compared the rates of melanoma among Swedish men in various industries over a 19 year period [29]. When reporting on melanomas at all body sites, that study found rates of melanoma among farmers (a group of outdoor workers considered to have high levels of sun exposure) were not increased overall; however, farmers experienced an excess of melanomas on the scalp, face and neck. Another Swedish study followed 389,739 men employed in the construction industry between 1971 and 1993 and found no difference in overall melanoma rates between those in the highest versus lowest categories of occupational sun exposure [30]. However, the rate of melanomas occurring on the head, face and neck among high sun‐exposure construction workers was twice that of the low sun‐exposure group.

Taken together, the body of evidence in the current literature supports the following statements:

1. At the population level, melanomas arising on different body sites have different associations with chronic sun exposure.
  - for melanomas arising on the head and neck, there is a strong association with chronic sun exposure.
  - for melanomas arising on the trunk there is a weak or no association with chronic sun exposure.
2. Overall, outdoor workers have a lower incidence and a lower mortality from melanoma than indoor workers.
3. When analysed by anatomical site, outdoor workers have a lower incidence of melanoma on habitually covered sites (e.g., the trunk), but a higher incidence of melanoma on sun‐exposed sites (e.g., face, scalp, ears, neck).

## 5 Medico‐Legal Considerations

The question of whether or not a melanoma has been caused by sun exposure received while a worker has been employed in a particular occupation is of relevance not only to workers seeking compensation but also to employers and insurance companies, frequently leading to litigation. It is important to note that the relationships described above are inferred from population data and are necessarily general in nature. For any given patient, it is impossible to deconstruct the totality of their past sun exposure into discrete components (viz. occupational vs. recreational vs. incidental) to be able to attribute the cause of their melanoma with certainty to one or other pattern of sun exposure. However, based on an assessment of the age of the patient at diagnosis, the anatomical site of the melanoma and the histological features of the tumour and surrounding skin (e.g., the presence or absence of a contiguous naevus or the presence or absence of epidermal solar damage), it is possible to determine whether occupational sun exposure played a greater or lesser role in causality. Generally speaking, melanomas arising on habitually sun‐exposed sites among older people subjected to high levels of sunlight during their working life would be likely to have been caused, at least in part, by work‐related exposure. In contrast, melanomas arising on habitually protected sites are much less likely to be due to occupational sun exposure, especially among younger people.

In the modern era, workplaces in many countries have adopted a pragmatic approach to sun protection, recognising that the risk attached to exposure is such that employers have a duty of care to protect their workers. While there is no European Union directive addressing natural UV sources, the Occupational Safety and Health (OSH) framework directive 89/391 has general principles concerning occupational risks, meaning employers have the responsibility to avoid risks to workers from natural UVR, assess risks that cannot be avoided and to implement protection measures [31]. In the USA, the Occupational Safety and Health Act requires employers to provide a safe and healthy workplace, which includes minimising the risk of sun exposure for outdoor workers [32]. Similarly, in Australia, WorkSafe Australia requires that persons in control of business undertakings must appropriately manage the risks of sun‐related disease and injury [33]. In the UK, a 1999 work health regulation requires employers to assess risks from UV radiation from natural sunlight [34]. Even if the effectiveness of such measures in protecting workers from melanoma may be questioned, the benefits in terms of protection from the development of other forms of skin cancer such as squamous cell carcinoma are more certain.

## 6 Conclusions

There is converging and concordant evidence from epidemiological, molecular, and pathological studies that most cutaneous melanomas are caused by sunlight or artificial sources of UV (e.g., from sunbeds). While the extant literature regarding the role of sun exposure received in occupational settings reports inconsistent findings, the studies are largely unhelpful in resolving the question since they are predicated on outdated notions of melanoma causality.

Large‐scale cohort studies are required to clarify the effects of occupational sun exposure with greater confidence. These studies will need to report risks for the various melanoma sub‐types and stratify for important factors such as anatomical site, age, and tumour‐related features. Until the results of such studies become available, the contribution of occupational sun exposure to melanoma development in any given patient will continue to require considered, personalised assessment.

## Author Contributions

D.C.W. critically reviewed the manuscript. G.J.W. conducted the literature searches, identified relevant studies, appraised and summarised study data, and drafted the manuscript. J.F.T. conceived the idea for the study and critically reviewed the manuscript.

## Funding

D.C.W. is supported by an Investigator Grant (2026567) from the Australian National Health and Medical Research Council.

## Ethics Statement

No ethics approval was required. Only publicly available data were accessed.

## Conflicts of Interest

He has received travel and conference support from conference organisers but not directly from industry. He has provided expert testimony in legal cases on the subject of occupational sunlight exposure and risk of melanoma. G.J.W.‐None. J.F.T. has received honoraria for advisory board participation from BMS Australia, MSD Australia, GSK and Provectus Biopharmaceuticals, and travel and conference support from GSK, Provectus Biopharmaceuticals and Novartis. He has provided expert testimony in legal cases on the subject of occupational sunlight exposure and risk of melanoma.

## Data Availability

All data analysed in this study are presented in the paper.
